# Development, Validation, and Utilization of a Luminex-Based SARS-CoV-2 Multiplex Serology Assay

**DOI:** 10.1128/spectrum.03898-22

**Published:** 2023-03-16

**Authors:** Daisy R. Roy, Troy J. Kemp, Katarzyna Haynesworth, Sarah A. Loftus, Ligia A. Pinto

**Affiliations:** a Vaccine, Immunity, and Cancer Directorate, Frederick National Laboratory for Cancer Research, Frederick, Maryland, USA; Barcelona Centre for International Health Research

**Keywords:** Luminex-based multiplex assay, SARS-CoV-2, serology, COVID-19

## Abstract

SARS-CoV-2 antibody testing is important for seroprevalence studies and for evaluating vaccine immune responses. We developed and validated a Luminex bead-based multiplex serology assay for measuring IgG levels of anti-SARS-CoV-2 antibodies against full-length spike (S), nucleocapsid (N), and receptor-binding domains (RBDs) of wild-type, RBD N501Y mutant, RBD E484K mutant, RBD triple mutant SARS-CoV-2 proteins, Sars-CoV-1, MERS-CoV, and common human coronaviruses, including SARS-CoV-2, OC43, 229E, HKU1, and NL63. Assay cutoff values, sensitivity, and specificity were determined using samples from 160 negative controls and 60 PCR-confirmed, SARS-CoV-2-infected individuals. The assay demonstrated sensitivities of 98.3%, 95%, and 100% and specificities of 100%, 99.4%, and 98.8% for anti-(S), -N, and -RBD, respectively. Results are expressed as IgG antibody concentrations in BAU/mL, using the WHO international standard (NIBSC code 20/136) for anti-SARS-CoV-2 IgG antibodies. When the multiplex assay was performed and compared with singleplex assays, the IgG antibody measurement geometric mean ratios were between 0.895 and 1.122, and no evidence of interference was observed between antigens. Lower and upper IgG concentration limits, based on accuracy (between 80% and 120%), precision (percent relative standard deviation, ≤25%), and sample dilutional linearity (between 75% and 125%), were used to establish the assay range. Precision was established by evaluating 24 individual human serum samples obtained from vaccinated and SARS-CoV-2-infected individuals. The assay provided reproducible, consistent results with typical coefficients of variation of ≤20% for all assays, irrespective of the run, day, or analyst. Results indicate the assay has high sensitivity and specificity and thus is appropriate for use in measuring SARS-CoV-2 IgG antibodies in infected and vaccinated individuals.

**IMPORTANCE** The SARS-CoV-2 pandemic resulted in the development and validation of multiple serology tests with variable performance. While there are multiple SARS-CoV-2 serology tests to detect SARS-CoV-2 antibodies, the focus is usually either on only one antigen at a time or multiple proteins from only one SARS-CoV-2 variant. These tests usually do not evaluate antibodies against viral proteins from different SARS-CoV-2 variants or from other coronaviruses. Here, we evaluated a multiplex serology test based on Luminex technology, where antibodies against multiple domains of SARS-CoV-2 wild type, SARS-CoV-2 mutants, and common coronavirus antibodies are detected simultaneously in a single assay. This Luminex-based multiplex serology assay can enhance our understanding of the immune response to SARS-CoV-2 infection and vaccination.

## INTRODUCTION

Coronavirus disease (COVID-19) is an infectious disease caused by the severe acute respiratory syndrome coronavirus 2 (SARS-CoV-2). SARS-CoV-2 genome sequence analysis has provided unprecedented opportunities for tracking variants. Compared with the original Wuhan strain, SARS-CoV-2 variants of concern (Alpha, Beta, Gamma, Delta, and Omicron) and other known variants (Eta, Kappa, Lambda, Epsilon, Zeta, Theta, and Mu) have been associated with severe disease or increased transmission (1–3). Licensed vaccines protect people from serious illness and death caused by COVID-19 (2). Protection against SARS-CoV-2 infection is mediated by functional, neutralizing antibodies specific to SARS-CoV-2. Hence, monitoring antibody responses to infection and immunogenicity of vaccines is critical as it provides important insight into the progression of regional outbreaks, rates of exposure/infection, and viral transmission dynamics. Given the evolution of SARS-CoV-2 over the last 3 years, the development of an agile and dynamic multiplex serology-based assay is important to quickly respond to and monitor immune responses to new viral variants.

SARS-CoV-2 belongs to the *Betacoronavirus* genus of the *Coronaviridae* (CoV) family, which is closely related to the severe acute respiratory syndrome (SARS) coronavirus and the Middle East respiratory syndrome CoV (MERS-CoV). Certain coronaviruses are common human pathogens; two types of alphacoronaviruses (229E and NL63) and two types of beta-coronaviruses (OC43 and HKU1) circulate in humans and cause the common cold. More pathogenic coronaviruses for humans include SARS-CoV-1, MERS-CoV, and now SARS-CoV-2, which likely emerged from animals to humans (4). The genome of CoVs (27 to 32 kb) is a single-stranded positive-sense RNA larger than any other RNA viruses. SARS-CoV-2 contains four structural proteins (spike [S], envelope [E], membrane [M], and nucleocapsid [N] proteins) and 16 nonstructural proteins (nsp1 to nsp16) (4).

The N-terminal part of the S protein (S1) contains a receptor-binding domain (RBD), which is the structural part of SARS-CoV-2 involved in viral attachment to host cells and immunity by specifically recognizing the angiotensin-converting enzyme 2 (ACE2) receptor. The total nucleotide sequence similarity between SARS-CoV-2 and SARS-CoV is approximately 79% and is about 50% between SARS-CoV-2 and MERS-CoV (5).

Here, we describe the development and validation of a 15-plex Luminex bead-based assay to measure the concentration of IgG antibodies specific to SARS-CoV-2 proteins and against six other coronaviruses. We evaluated the assay performance characteristics to detect the presence of antibodies directed to the S, N, and RBD (wild type [WT] and variants) proteins of SARS-CoV-2 in SARS-CoV-2-infected and vaccinated individuals.

## RESULTS

### Selection of appropriate proteins for the Luminex multiplex assay.

Multiple SARS-CoV-2-related proteins were evaluated and compared as antigen targets for the Luminex serological multiplex assay by using a standard and a subset of serum samples collected from participants who were infected with and recovered from SARS-CoV-2 and SARS-CoV-2 seronegative samples (collected prior to December 2019) for IgG detection. SARS-CoV-2 S protein RBD constructs were developed by Mount Sinai (Icahn School of Medicine at Mt. Sinai, New York, NY) and designated with an “M” in the protein name or by the Ragon Institute of Massachusetts General Hospital (MGH), Massachusetts Institute of Technology (MIT), and Harvard (Boston, MA) and designated with an “R” in the protein name; these constructs were used for RBD WT and variant protein constructions.

**FIG 1:**
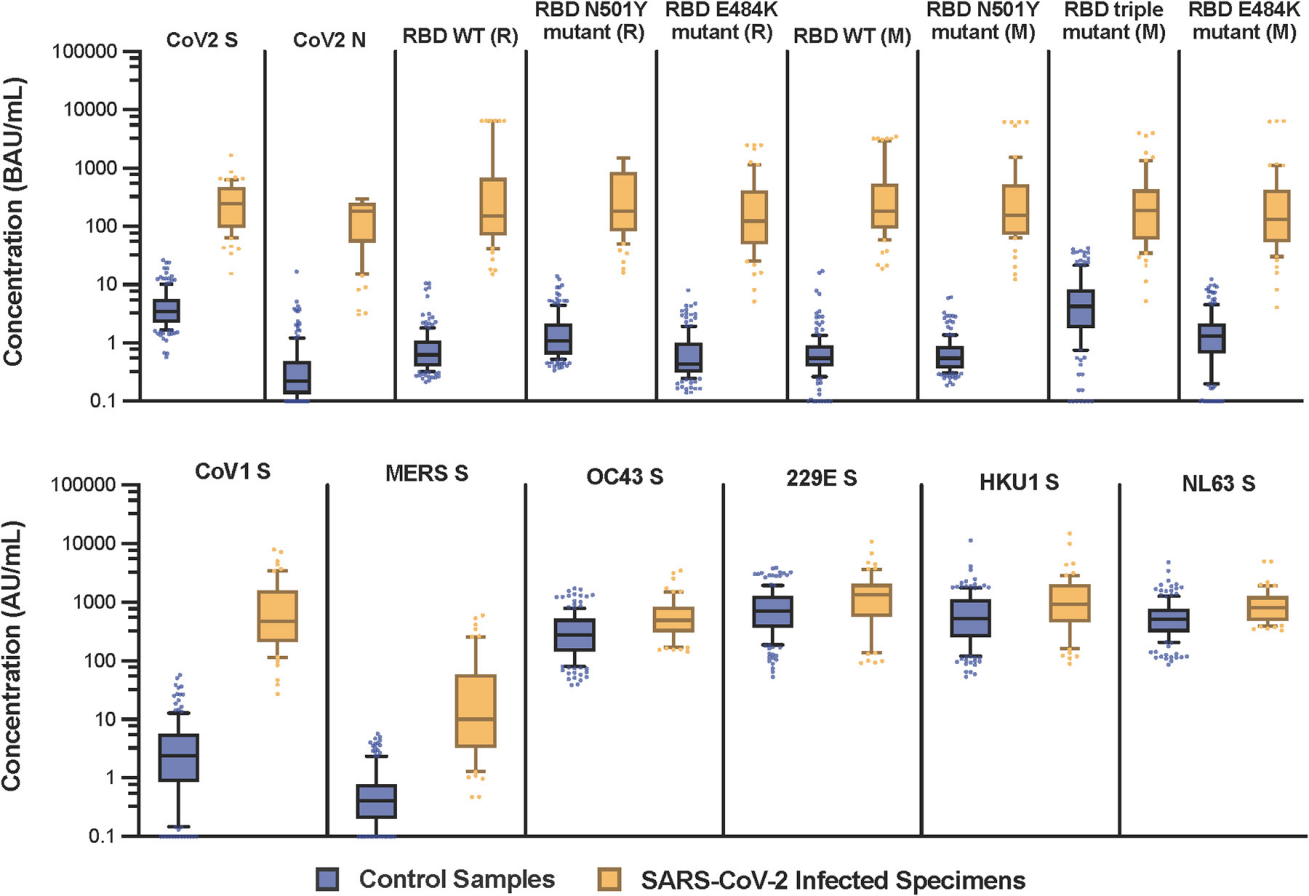
Analysis of samples from SARS-CoV-2-negative (serum or plasma collected prior to December 2019) and SARS-CoV-2-infected individuals by the Luminex multiplex assay. Protein sequences were associated with Mount Sinai (M) and Ragon (R). The concentration unit for MERS S is IU/mL.

The short form of the SARS-CoV-2 N protein and the Ragon (R) RBD triple mutant showed poor discrimination between serum collected from participants who were infected with and recovered from SARS-CoV-2 and control (SARS-CoV-2 seronegative) serum compared with other SARS-CoV-2 proteins (data not shown). Consequently, these two proteins were excluded from the assay, and we selected the full-length SARS-CoV-2 N protein, along with WT and mutant RBD protein from Ragon (R) and Mount Sinai (M), for the multiplex assay. SARS-CoV-1, MERS-CoV (MERS), and seasonal CoV N-based antigens gave high background signal in the diluent alone and had a narrow dynamic range of the standard compared with the S proteins. These results supported the use of mammalian-expressed S proteins for SARS-CoV-1, MERS, and seasonal CoVs. Good discretion between the 160 seropositive and 60 seronegative samples was observed on all SARS-CoV-2 proteins, SARS-CoV-1 S, and MERS S (Fig. 1).

### Multiplexing interference.

The possibility of interference between related proteins in the multiplex assay was evaluated by comparing four serum samples collected from participants who were infected with and recovered from SARS-CoV-2, a serology reference standard, and three quality control (QC) samples in the 15-plex assay against 15 single assays. Table S1 and Fig. S1 in the supplemental material show the analysis of percent error and geometric mean ratios (GMR) between the data generated in singleplex and 15-plex assays. GMR fell within the range of 0.895 to 1.122 for all assays, suggesting that multiplexing did not alter the concentration of IgG antibodies for all assays.

### Assay specificity assessment.

Analytical specificity of the capture proteins or their degree of cross-reactivity was investigated using protein-specific monoclonal antibodies (RBD-binding mAb109, S2-binding mAb112, and mAb118 [Vaccine Research Center, NIAID]; 40150-D001, 40150-D003, and 40143-R001 [Sino Biologicals]) reactive to SARS-CoV-2 and SARS-CoV-1 proteins. Monoclonal antibodies targeting OC43, NL63, HKU1, 229E, and MERS S proteins were not assessed. The binding specificity of the monoclonal antibodies from BEI and Sino Biologicals showed antigen-specific binding. The S2 antibodies bound only to the full-length spike protein of SARS-CoV-2 and SARS-CoV-1, and the RBD antibody bound only to RBD of SARS-CoV spike proteins and not the MERS or seasonal coronavirus proteins. Acceptable specificity results were observed in evaluations of the commercially available monoclonal antibodies, as shown in Fig. S2A and B in the supplemental material.

Similarly, specificity was evaluated using immunodepleting serum for SARS-CoV-2 and OC43 CoV S proteins. The OC43 CoV S proteins were used for immunodepletion because OC43 is a betacoronavirus among the four seasonal coronaviruses and is responsible mostly for mild respiratory symptoms. The Luminex serology assay standard was incubated separately with SARS-CoV-2 S protein, OC43 S protein, or assay diluent for 2 hours (h) prior to analysis. The percentage of IgG depletion was calculated by normalizing the median fluorescence intensity (MFI) in the presence of competitors relative to the MFI observed from the assay diluent only (control condition in the absence of specific protein). A summary of the data is shown in Table S2. Using the SARS-CoV-2 S protein as the competitor, ≥80% reduction of the specific antibody concentration relative to the control was observed in the SARS-CoV-2 and SARS-CoV-1 assays. Adding the heterologous OC43 S as a competitor resulted in <10% inhibition for all proteins, except structurally similar SARS-CoV-1 S, where some level of cross-reactivity was expected and observed.

### Determination of optimal reagent concentrations and protocol parameters.

We tested three different protein concentrations (50, 5, and 0.5 μg of protein per 1.25 × 10^6^ beads) and two secondary antibody concentrations (1:125 and 1:62.5). We found that S CoV proteins at 5 μg per 1.25 × 10^6^ beads, N SARS-CoV-2 protein at 0.5 μg per 1.25 × 10^6^ beads, and RBD WT and mutant SARS-CoV-2 proteins at 5 μg per 1.25 × 10^6^ beads showed good linearity, high specific signals, and good signal ratio between positive and negative samples. A secondary antibody concentration obtained with a 1:125-fold dilution gave a high specific signal with low background.

Different concentrations of reagents and incubation times were also assessed to optimize and standardize assay conditions. Precoupled beads were adjusted to a working concentration of 2,000 beads per well (data not shown) after testing 4,000, 2,000, 1,000, and 500 beads per well. We chose 30 minutes (min) of incubation time at room temperature (i.e., 18 to 25°C) as the optimal condition that achieved acceptable assay sensitivity and robust assay performance after comparing assays performed at 30 min and 2 h at room temperature to overnight at 2 to 8°C. All the optimal reagent concentrations and conditions chosen for the Luminex multiplex assay are shown in Table S3 in the supplemental material.

### Assay sensitivity and specificity.

The Luminex-based SARS-CoV-2 multiplex serology assay is a quantitative test, and the analyte concentrations of each sample are reported as units per milliliter that are further defined in Table 1. The cutoff concentrations for each protein were determined based on the mean concentration plus five standard deviations of 160 serum or plasma samples collected prior to the pandemic (prior December 2019), and thus, they were presumably unexposed and SARS-CoV-2 seronegative by other assays, such as enzyme-linked immunosorbent assays (ELISAs). With this cutoff, three samples from the SARS-CoV-2-negative group tested positive for SARS-CoV-2 RBD WT (R), with a specificity of 98.1% (*n* = 157/160). Sensitivity and specificity data for each of the assays within the multiplex are demonstrated in Table 2. These assays show a range of sensitivity from 83.3% with the RBD triple mutant (M) to a sensitivity of 100% with the RBD WT and N501Y mutants (R and M). All proteins demonstrated a specificity over 98.1%. Sensitivity or positive predictive agreement (PPA) is the proportion of subjects with the target condition in whom the test is positive. Specificity, or negative predictive agreement (NPA), is the proportion of subjects without the target condition in whom the test is negative (6).

**TABLE 1:** Information on the CoV proteins included in the Luminex multiplex serology assay^*a*^

Recombinant protein	Region (aa)	Expression system	Full name of protein	Reporting units
SARS-CoV-2-S	14–1213	Expi293F cells	SARS-CoV-2 S protein	BAU/mL
SARS-CoV-2-N	1–419	E. coli BL21(DE3)	SARS-CoV-2 N protein	BAU/mL
SARS-CoV-2-RBD (R)	319–529	Expi293F cells	SARS-CoV-2 RBD	BAU/mL
SARS-CoV-2-RBD (N501Y) mutant (R)	318–529	Expi293F cells	SARS-CoV-2 RBD (B.1.1.7)	BAU/mL
SARS-CoV-2-RBD (E484K) mutant (R)	318–529	Expi293F cells	SARS-CoV-2 RBD (E484K)	BAU/mL
SARS-CoV-2-RBD (M)	319–541	Expi293F cells	SARS-CoV-2 RBD	BAU/mL
SARS-CoV-2-RBD (N501Y) mutant (M)	319–541	Expi293F cells	SARS-CoV-2 RBD (B.1.1.7)	BAU/mL
SARS-CoV-2-RBD (K417N/E484K/N501Y) triple mutant (M)	319–541	Expi293F cells	SARS-CoV-2 RBD (B.1.351)	BAU/mL
SARS-CoV-2-RBD (E484K) mutant (M)	319–541	Expi293F cells	SARS-CoV-2 RBD (E484K)	BAU/mL
SARS-CoV-1-S	14–1190	Expi293F cells	SARS-CoV-1 S protein	AU/mL
MERS-S	18–1291	Expi293F cells	MERS S protein	IU/mL
HCoV-OC43	15–1278	Expi293F cells	Human coronavirus OC43 S protein	AU/mL
HCoV-229E	16–1110	Expi293F cells	Human coronavirus 229E S protein	AU/mL
HCoV-HKU1	14–1276	Expi293F cells	Human coronavirus HKU1 S protein	AU/mL
HCoV-NL63	15–1291	Expi293F cells	Human coronavirus NL63 S protein	AU/mL

a Mount Sinai (M) and Ragon (R) proteins differ in their C-terminal tags and construct signal peptides.

**TABLE 2:** Performance characteristics of the quantitative Luminex multiplex serology assay^*a*^

Assay^*b*^	Assay cutoff concentration	Sensitivity (% [PPA])	Specificity (% [NPA])	PPV (%)	NPV (%)
CoV-2 S	26.9 BAU/mL	98.3 (59/60)	100 (160/160)	100.0	99.4
CoV-2 N	8.2 BAU/mL	95.0 (57/60)	99.4 (159/160)	98.3	98.1
CoV-1 S	49.2 AU/mL	95.0 (57/60)	98.8 (158/160)	96.6	98.1
MERS CoV S	6.3 IU/mL	58.3 (35/60)	100 (160/160)	100.0	86.5
OC43 CoV S	2,118.0 AU/mL	5.00 (3/60)	100 (160/160)	100.0	73.7
229E CoV S	5,037.0 AU/mL	3.33 (2/60)	100 (160/160)	100.0	73.4
HKU1 CoV S	6,118.0 AU/mL	3.33 (2/60)	99.4 (159/160)	66.7	73.3
NL63 CoV S	3,603.0 AU/mL	3.33 (2/60)	99.4 (159/160)	66.7	73.3
RBD WT (R)	8.9 BAU/mL	100 (60/60)	98.1 (157/160)	95.2	100.0
RBD mutant N501Y (R)	12.6 BAU/mL	100 (60/60)	99.4 (159/160)	98.4	100.0
RBD mutant E484K (R)	6.1 BAU/mL	98.3 (59/60)	99.4 (159/160)	98.3	99.4
RBD WT (M)	11.0 BAU/mL	100 (60/60)	98.8 (158/160)	96.8	100.0
RBD mutant N501Y (M)	5.0 BAU/mL	100 (60/60)	98.8 (158/160)	96.8	100.0
RBD triple mutant (M)	52.1 BAU/mL	83.3 (50/60)	100 (160/160)	100.0	94.1
RBD mutant E484K (M)	12.7 BAU/mL	96.7 (58/60)	100 (160/160)	100.0	98.8

a Shown here are sensitivity and specificity estimates and the ratio of responsive samples for PPA and NPA, calculated using the Wilson score method. Positive predictive value (PPV) and negative predictive value (NPV) were estimated using combined PPA and combined NPA, respectively (6). The assay cutoff concentrations for each protein were determined based on the mean concentration plus five standard deviations of 160 serum or plasma samples collected prior to the pandemic, prior December 2019 and thus, presumably unexposed and SARS-CoV-2 seronegative by other assays, such as ELISAs.

b Protein sequences were associated with Mount Sinai (M) and Ragon (R).

**FIG 2:**
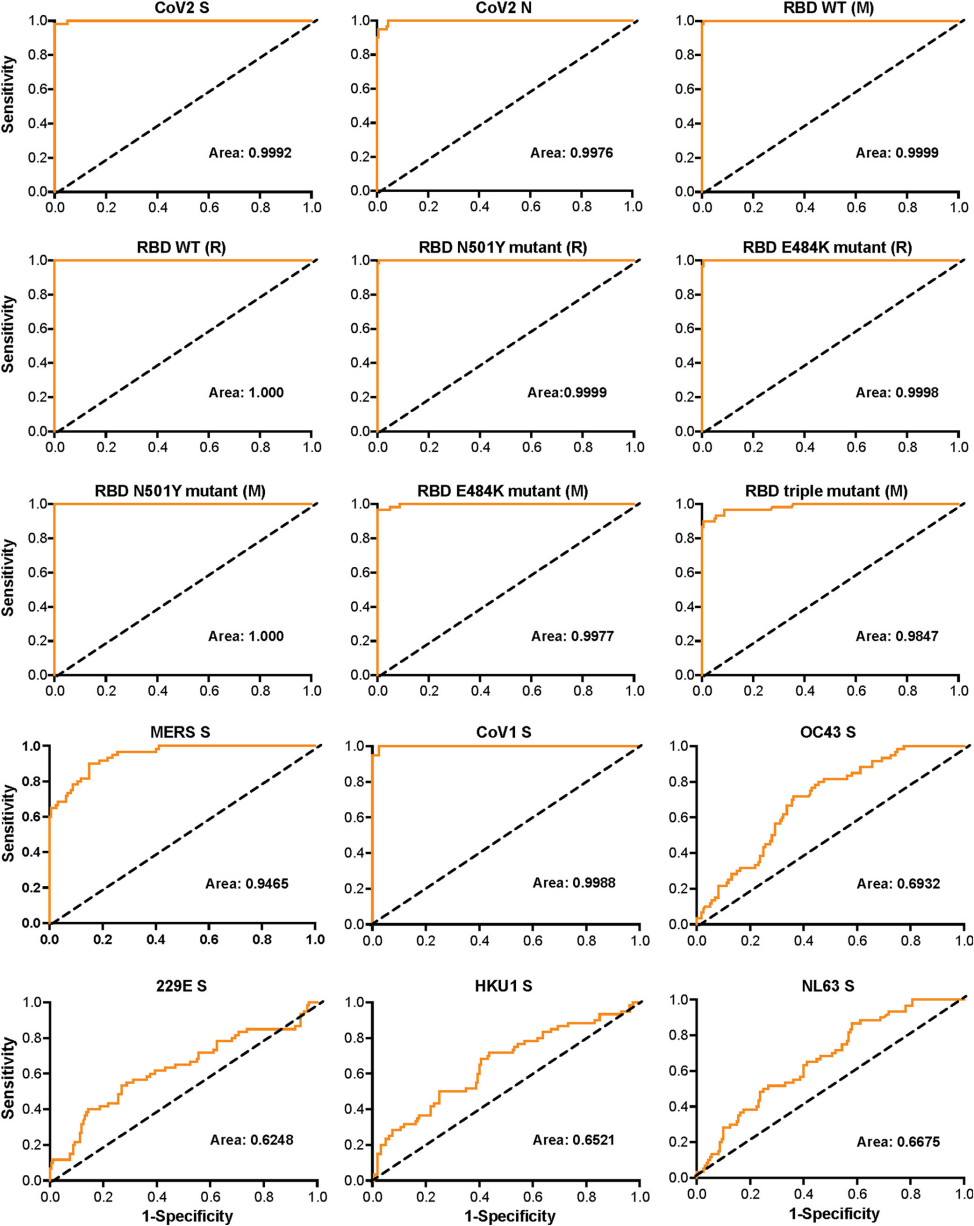
ROC curves for the multiplex Luminex-based serology assay based on the defined assay cutoffs to predict SARS-CoV-2 positivity in serum and plasma samples. Values represent the area under the curve (AUC). Protein sequences were associated with Mount Sinai (M) and Ragon (R).

Sensitivity and specificity were calculated for all proteins within the multiplex using a receiver operating characteristic (ROC) analysis for the SARS-CoV-2-infected participants (<7 weeks) (Fig. 2 and Table 2). The ROC curve analysis, based on the results of serum samples collected from 60 SARS-CoV-2-infected participants and 160 serum or plasma samples collected prior to December 2019 (SARS-CoV-2 negative, yet presumed positive for seasonal coronaviruses), calculated the area under the ROC curve (AUC), a commonly used measure for the overall accuracy of a marker. Based on the performance characteristics (≥83.3% sensitivity and ≥98.8% specificity) and AUC values of each SARS-CoV-2 assay, the data demonstrate that the Luminex multiplex serology assay is very well suited for highly sensitive characterization and quantification of humoral responses to SARS-CoV-2.

### Upper and lower limits of quantification.

SARS-CoV-2-positive human serum samples with expected concentrations near the high- and low-calibration points were run on 3 days by two analysts where two replicates were run each day. Table 3 shows the dilution-adjusted lower limit of detection (LLOD), lower limit of quantitation (LLOQ), and upper limit of quantitation (ULOQ) by protein and fold range (the ratio of the ULOQ and the LLOQ) of the standard curve for each protein. The LLOQ reflects the lowest measurable IgG concentration in the highest concentration of the sample matrix.

**TABLE 3:** Final assay range and calculated LLOD, LLOQ, and ULOQ (dilution adjusted) for all the capture proteins

Assay^*a*^	Units	LLOD	LLOQ	ULOQ	Fold difference (ULOQ/LLOQ)
CoV-2 S	BAU/mL	3.5	11.5	442.5	38.5
CoV-2 N	BAU/mL	1.1	2.3	63.1	27.4
CoV-1 S	AU/mL	3.9	8.1	1386	171.1
MERS CoV S	IU/mL	0.7	0.8	41.4	51.8
OC43 CoV S	AU/mL	3.9	33.1	689.4	20.8
229E CoV S	AU/mL	3.9	20.3	2638.3	130.0
HKU1 CoV S	AU/mL	3.9	28.8	1,390.6	48.3
NL63 CoV S	AU/mL	3.9	47	1,392.4	29.6
RBD WT (R)	BAU/mL	1.3	12.7	87.9	6.9
RBD mutant N501Y (R)	BAU/mL	1.8	14.9	878.5	59.0
RBD mutant E484K (R)	BAU/mL	1.1	9.5	338.8	35.7
RBD WT (M)	BAU/mL	1.5	13.3	237.1	17.8
RBD mutant N501Y (M)	BAU/mL	1.6	12.4	171.3	13.8
RBD triple mutant (M)	BAU/mL	1.5	13.9	316.8	22.8
RBD mutant E484K (M)	BAU/mL	1.1	9.2	278.2	30.2

a Protein sequences were associated with Mount Sinai (M) and Ragon (R).

**FIG 3:**
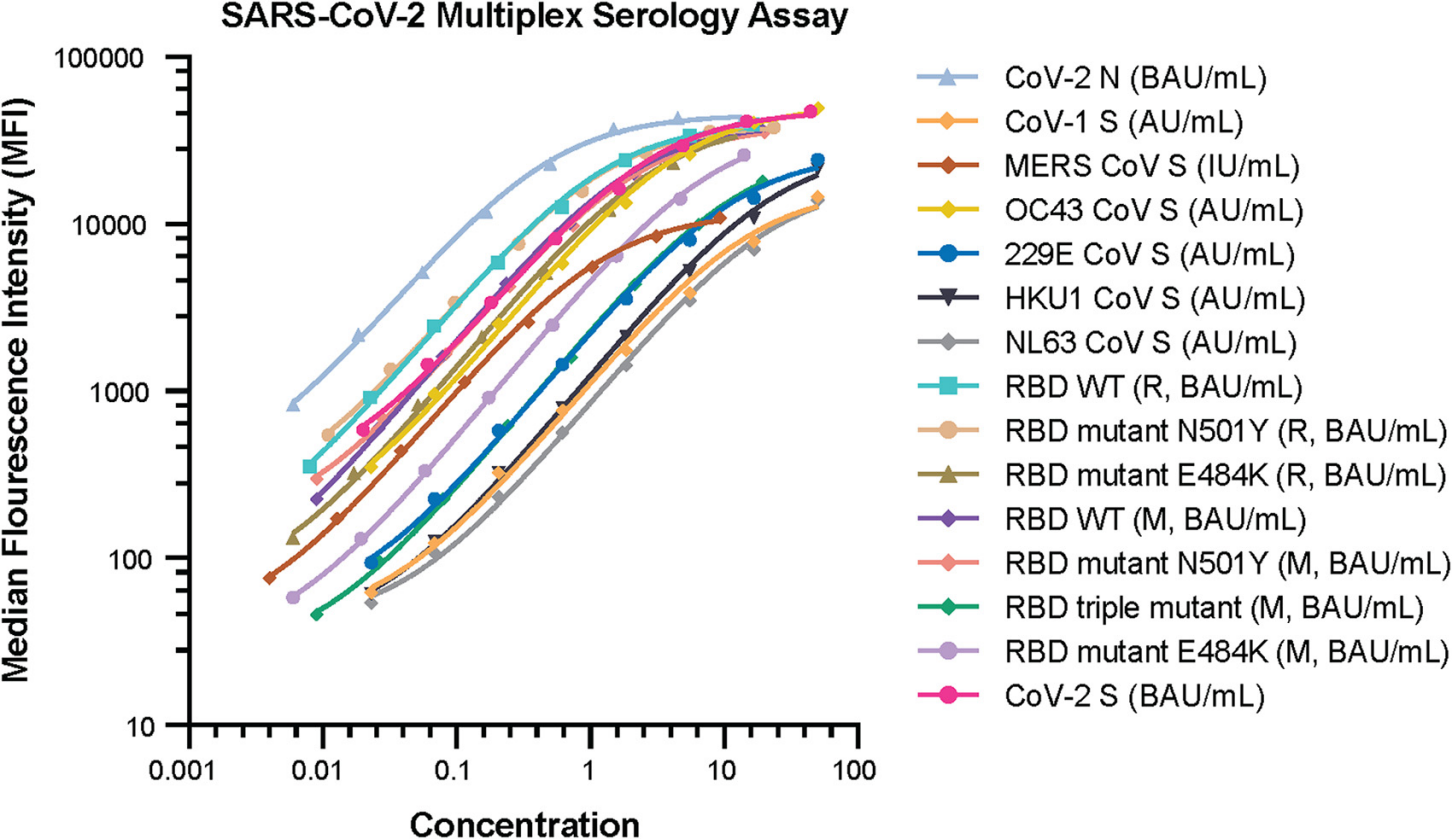
Dynamic ranges of anti-CoV IgG standard serum for each of the 15 CoV proteins. MFI signal data for the standard curve are on the *y* axis, and specific IgG antibody concentration data are on the *x* axis. The figure legend indicates the concentration unit for each CoV protein. Protein sequences were associated with Mount Sinai (M) and Ragon (R).

The standard, which has assigned IgG antibody concentrations for all 15 CoV proteins, was used as the reference standard in the 15-plex multiplex serology assay. Eight 3-fold dilutions of Luminex standard serum were performed and used in the assay. Figure 3 shows the Luminex standard dilution profiles for each of the 15 CoV proteins as fitted by a five-parametric regression model. Table S5 in the supplemental material lists the MFI values and concentrations for the Luminex standard for each of the 15 CoV proteins.

### Precision.

Estimates of assay variability due to intraday, interday, or analyst were calculated over 20 replicates. The intra- and interday or analyst repeatability of each assay demonstrated a coefficient of variation (CV) of ≤15.6% as shown in Table 4. These results confirmed that the Luminex-based multiplex serological assay is highly reproducible.

**TABLE 4:** Intra/interday and interanalyst precision^*a*^

Assay^*b*^	No. of measurements	% RSD				ICC
		Mean intraday	Mean interday	Mean analyst	Total	
CoV-2 S	480	11.6	2.8	5.5	13.1	99.3
CoV-2 N	240	11.6	5.1	4.1	13.3	98.4
CoV-1 S	480	13.8	1.8	2.7	14.1	99.1
MERS CoV S	478	15.6	4.8	2.7	16.6	99.1
OC43 CoV S	480	11.3	3.4	5.2	12.9	99.1
229E CoV S	480	10.8	3.4	3.9	12.0	99.4
HKU1 CoV S	480	11.7	1.5	5.2	12.9	98.9
NL63 CoV S	480	11.3	3.3	3.9	12.4	99.1
RBD WT (R)	480	11.7	6.4	3.4	13.8	99.3
RBD mutant N501Y (R)	480	11.7	5.1	4.9	13.7	99.3
RBD mutant E484K (R)	480	11.4	3.8	3.6	12.6	99.6
RBD WT (M)	480	12.1	3.4	3.6	13.1	99.3
RBD mutant N501Y (M)	480	11.8	4.2	3.6	13.0	99.3
RBD triple mutant (M)	480	11.6	4.1	2.8	12.7	99.6
RBD mutant E484K (M)	480	11.5	3.0	3.2	12.3	99.6

a A percent error ≤25% threshold was used to define at which concentrations the assay may be deemed acceptable. Data include replicates and % relative standard deviation (RSD) or CV and intraclass correlation (ICC), which is a measure of true variance to total observed variance.

b Protein sequences were associated with Mount Sinai (M) and Ragon (R).

### Accuracy.

**FIG 4:**
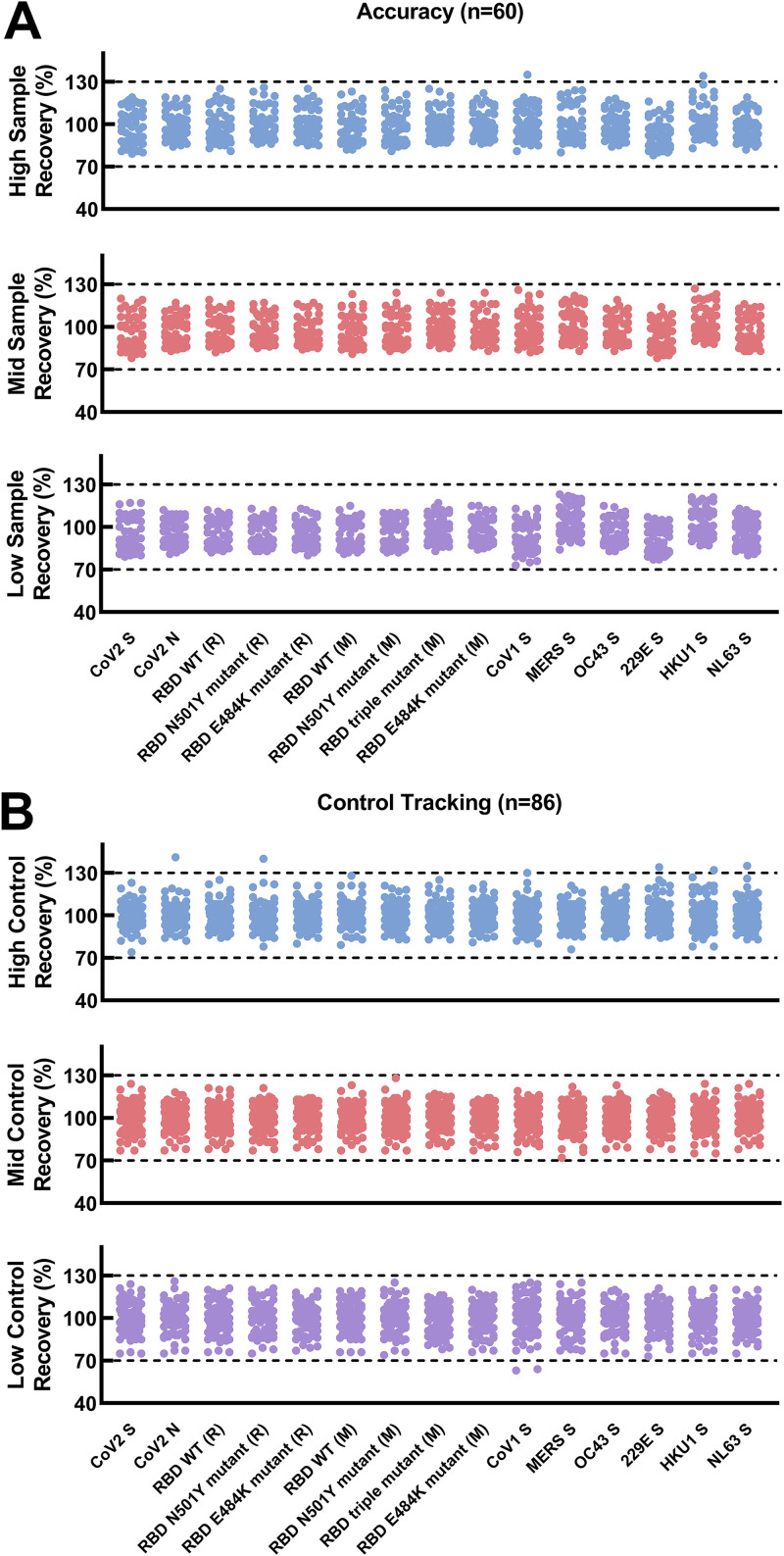
Evaluation of the accuracy and control tracking of the multiplex assay. (A) Accuracy was evaluated by comparing the observed values to the expected values of a single sample that was diluted to three distinct levels. Three levels of assay control (B) were also evaluated. Each graph shows the percent recovery of the IgG concentration as calculated by the observed IgG concentration over expected IgG concentration (*y* axis) of high, mid, and low sera across each CoV protein (*x* axis). The 70% to 130% limits are indicated by dotted black lines. Protein sequences were associated with Mount Sinai (M) and Ragon (R).

To determine accuracy, we tested three SARS-CoV-2 seropositive samples spanning the linear range of the assays across multiple runs. These samples are known to be positive for antibodies to SARS-CoV-2, SARS-CoV-1, MERS, and all four of the seasonal coronaviruses. Measured concentrations of each sample were compared with the expected concentrations based on the nominal value (mean of the calculated concentrations) determined during assay verification. In total, 60 results per analyte were used to calculate the accuracy of the assay. Accuracy was determined to be between 93.4% and 100% (Fig. 4A).

The performance and robustness of the 15-plex assay were monitored during validation testing with QC samples (QCs), which are included on each assay plate and tested in all runs (*n* = 86). QC results were collected from each assay run and demonstrated <20% variability over time (data not shown). Trending analysis of QC graphs are shown in Fig. 4B. Measured concentrations of QCs were compared with the expected concentrations based on the nominal value (mean of the calculated concentrations across runs) determined during assay verification. Trending analysis showed that the assay had been performing in a consistent manner (within the 70% to 130% limits indicated by dashed lines in Fig. 4B) throughout the 3-month testing period of validation.

### Linearity.

**FIG 5:**
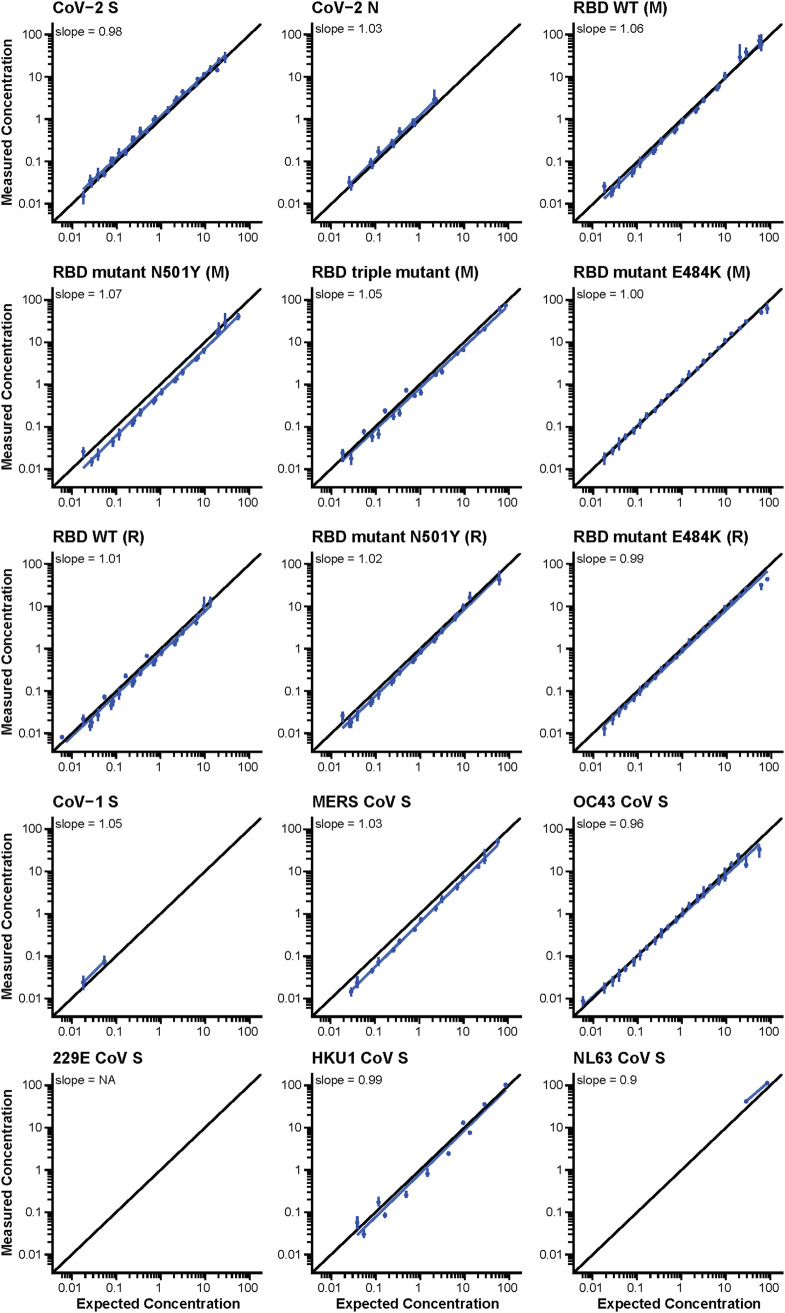
The serially diluted human SARS-CoV-2-positive samples shown here have a percent error of ≤50% and % relative standard deviation (RSD) of ≤30%. The 229E sample levels were <LLOQ, and there was no correlation. Protein sequences were associated with Mount Sinai (M) and Ragon (R). Note the concentration units for each CoV protein are listed in Table 1.

Four human SARS-CoV-2-positive samples were tested to evaluate the overall linearity of the assay. The difference between the observed and expected IgG concentration was calculated as the percent error. Depending on the protein, the lower and upper dilutional linearity limits of human serum samples fell within the working range of the assay (Table 5; Fig. 5).

**TABLE 5:** Linearity statistics for each assay, including minimum and maximum correlation coefficients and slope^*a*^

Assay^*b*^	Assay abbreviation	Minimum r	Maximum r	Slope
CoV-2 S	CoV2 S	0.993	1.00	0.980
CoV-2 N	CoV2 N	0.998	0.999	1.03
CoV-1 S	CoV1 S	NA	NA	1.05
MERS CoV S	MERS S	0.999	1.00	1.03
OC43 CoV S	OC43 S	0.987	1.00	0.960
229E CoV S	229E S	NA	NA	NA
HKU1 CoV S	HKU1 S	1.00	1.00	0.990
NL63 CoV S	NL63 S	NA	NA	0.900
RBD WT (R)	Ragon RBD	0.996	1.00	1.01
RBD mutant N501Y (R)	Ragon RBD UK	0.998	0.999	1.02
RBD mutant E484K (R)	Ragon RBD E484	0.969	1.00	0.990
RBD WT (M)	M RBD	0.986	0.999	1.06
RBD mutant N501Y (M)	M RBD UK	0.994	0.996	1.07
RBD triple mutant (M)	M RBD SA	0.999	1.00	1.05
RBD mutant E484K (M)	M RBD E484K	0.986	1.00	1.00

a Each assay that met acceptance criteria of percent error of ≤50% and % RSD of ≤30% for samples was used to calculate r. If the maximum number of samples that passed the threshold for r is ≤3, the value is reported as not applicable (NA). 229E samples do not meet the percent error and % RSD thresholds, and hence, r and slope are reported as NA.

b Protein sequences were associated with Mount Sinai (M) and Ragon (R).

## DISCUSSION

Validation experiments determined assay accuracy, precision, linearity, and specificity. The SARS-CoV-2 multiplex immunoassay was found to have an optimal specificity for IgG detection, shown by a PPV of 100% for SARS-CoV-2 S and higher than 98% for SARS-CoV-2 N, SARS-CoV-2 RBD WT, and mutant proteins, which indicates that the assay would be a valuable tool for seroprevalence studies and vaccine response analyses (7). The specificity of the assay demonstrated that only CoV-specific IgG antibodies were detected, which suggests that antigenic epitopes on the CoV proteins are not adversely affected by the conjugation reaction. Comparison of the IgG concentrations measured by the singleplex and 15-plex assays demonstrated that multiplexing did not appear to alter the concentration of IgG antibodies for the evaluated CoV protein. For standardization, the WHO international and U.S. serology standards for anti-SARS-CoV-2 immunoglobulins were used. The Luminex multiplex serology assay was found to give reliable results at both low and high levels as observed in Fig. 4.

One potential limitation of the study relates to the SARS-CoV-2 variants evaluated during the validation testing. The SARS-CoV-2 seropositive samples tested were collected within the United States between April 2020 and April 2021. Unfortunately, viral sequencing was not performed for any of the participants’ infections, so we cannot guarantee the type of variant to which the participants were exposed. However, based on the collection time points and strain predominance in the United States, the participants were most likely infected with the original or alpha strain. Since SARS-CoV-2 evolves continuously, sera from individuals infected with emerging variants of the virus need to be assessed to evaluate their impact on assay performance. The multiplex SARS-CoV-2 assay provides a flexible platform for future additions of antigens for SARS-CoV-2 variants as necessary. Another limitation of the study involves the seasonal coronavirus detection. Understanding that antibody responses to the seasonal coronaviruses are fairly ubiquitous in the population, it makes it very difficult to assess specificity and to establish reliable assay cutoffs, so additional evaluation will be needed to establish firm assay cutoffs.

There are several commercial and in-house developed multiplex assays that were developed from bead-based (Luminex) or electrochemiluminescence plate-based (MSD) platforms (8–11). Although we have not performed any formal comparisons with these other assays, our performance characteristics are similar when compared indirectly. Even though the assays may have similar performance characteristics, it is still very critical to use international and national standards to allow for comparisons between results.

Current vaccines distributed in the United States induce antibodies to S proteins (2). Most individuals appear to produce antibodies to both S and N proteins of SARS-CoV-2 after infection. Furthermore, anti-N IgG appears to have a shorter duration than anti-S IgG or –RBD IgG (12–16) which could result in an underestimation of the seroprevalence when one uses only N-based serological assays. A SARS-CoV-2 multiplex serology assay has the advantage of being used to monitor antibody responses to each of the following proteins: S, N, and RBD of SARS-CoV-2. In conclusion, a 15-plex immunoassay for the simultaneous measurement of IgG antibodies against 15 CoV proteins in human serum samples was developed, validated, and found adequate for use in seroprevalence and vaccine immunogenicity studies.

## MATERIALS AND METHODS

### Samples.

Human serum or plasma samples were obtained through Biodefense and Emerging Infections Research Resources Repository (BEI), CDC, Vitalant Research Institute, Arizona State University, Icahn School of Medicine at Mount Sinai, Northwestern University, BioIVT, and Boca Biolistics. The samples were collected under approved protocols. Additional samples were collected from Occupational Health Services, Frederick National Laboratory for Cancer Research, Frederick, Maryland, under the Research Donor Protocol OH99CN046. In total, 208 serum and plasma samples were collected prior to December 2019 and selected for testing as SARS-CoV-2-seronegative samples. For SARS-CoV-2-seropositive samples, 81 serum samples were collected from participants who were infected with and recovered from SARS-CoV-2, and the sample collection date occurred between 17 and 46 days post-symptom onset. Also, 16 serum samples were collected from SARS-CoV-2-vaccinated participants. Infection from SARS-CoV-2-infected participants was confirmed via a molecular test at the collection site. The samples were also tested with our in-house-developed SARS-CoV-2 spike ELISA to screen the samples for IgG reactivity (6).

### Luminex bead-based multiplex serology assay.

Serum samples were tested for a SARS-CoV-2-specific immune response using the Luminex bead-based multiplex serology assay. The multiplex assay measures antibody responses to 15 unique CoV proteins. The Luminex assay uses distinct fluorescent microspheres (bead) onto which each CoV protein is individually coupled and eventually mixed to create a multiplex set. Antibodies in the sample bind to the protein-coupled bead and anti-human IgG antibodies conjugated to phycoerythrin (PE). The Luminex dual-laser detection instrument Luminex FlexMap 3D with the xPONENT 4.3 software determines the target protein based on the bead and measures the antibody response based on the magnitude of the PE signal.

### Proteins (antigens).

Fifteen CoV proteins (Protein Expression Laboratory [PEL], Frederick National Laboratory for Cancer Research [FNL], Frederick, MD) were used as antigens in the multiplex serology assay (Table 1). SARS-CoV-2 S protein RBD constructs were developed by Mount Sinai (Icahn School of Medicine at Mt. Sinai, New York, NY) and designated with an “M” in the protein name or by the Ragon Institute of Massachusetts General Hospital (MGH), Massachusetts Institute of Technology (MIT), and Harvard (Boston, MA) and designated with an “R” in the protein name; these constructs were used for RBD WT and variant protein constructions. Briefly, all the proteins except nucleocapsid proteins were expressed in Expi293F cells. Nucleocapsid proteins were expressed in Escherichia coli and grown in Dynamite broth. The proteins were purified by tangential flow filtration, immobilized metal affinity chromatography, desalting, and/or size exclusion chromatography (SEC). The proteins were further quality controlled with the following techniques: SDS-PAGE, SEC, and electrospray ionization-mass spectrometry (ESI-MS). The proteins are available upon request for SeroNet members and technical service agreements.

### Protein coupling.

Proteins were coupled to carboxylated microspheres (Luminex, Austin, TX) using a Luminex coupling kit (catalog number 40-50016) according to the manufacturer’s instructions, with slight modifications, as follows: the number of coupled beads was scaled up to 25.0 × 10^6^ beads/reaction; the buffer volume was increased to 2 mL during all wash steps, including the final protein incubation step; and the Luminex wash buffer was replaced with phosphate-buffered saline (PBS). Luminex beads were counted for quality checking using a cell counter as per the vendor instructions in the Luminex Xmap Cookbook. However, as the alternate approach from the cookbook, functional testing was employed to verify the performance of the protein-coupled beads.

### Multiplex serology assay standard and controls.

The multiplex serology assay standard was developed by pooling serum from three participants who were infected with and recovered from SARS-CoV-2 infection and showed reactivity (based on median fluorescence intensity [MFI] of ≥1,000) with all 15 proteins during screening. The controls were contrived from the standard by diluting the standard with a pool of seronegative serum. Controls were identified at high, mid, and low ranges of the standard curve, and their expected ranges were established during validation.

### Multiplex assay procedure.

Samples were heat inactivated for 30 to 60 min at 56°C as a standard precaution to minimize the risk of SARS-CoV-2 transmission. All test serum samples—standard and control—were diluted in PBS containing 1% bovine serum albumin (BSA), and 50 μL was added to the respective wells of the plate. The assay has an 8-point, 3-fold-dilution standard curve with a 20-fold starting dilution that was calibrated to the international reference standard (for each of the following levels of standard, the dilution factor is listed: STD1, 1/20; STD2, 1/60; STD3, 1/180; STD4, 1/540; STD5, 1/1,620; STD6, 1/4,860; STD7, 1/14,580; and STD8, 1/43,740). Test samples and controls were tested at four 3-fold dilutions with a 50-fold starting dilution (the dilution factor for each level of sample and control is as follows: dilution 1, 1/50; dilution 2, 1/150; dilution 3, 1/450; and dilution 4, 1/1,350). Controls were run on every plate to monitor the assay performance. Samples that fell above the assay range were retested at higher dilutions. Then, 50 μL of the bead mixture (solution containing each of the 15 protein-coupled beads in PBS containing 1% BSA) was added to each well, and the plate was incubated and shaken in the dark for 30 min. Plates were washed to remove the unbound isotype antibody and were tread using a Luminex analyzer. For the wash step, plates were placed on a magnetic plate separator, and R-phycoerythrin-labeled goat anti-human IgG secondary antibody (Jackson ImmunoResearch; catalog number 109-116-098) was applied to the wells and allowed to associate with complexed protein-antibody for 30 min in the dark with shaking. The samples were run on a Luminex FlexMap 3D instrument with the xPONENT 4.3 software. Results were expressed as MFI, and IgG antibody concentrations were calculated from the interpolation of sample MFI values to the reference standard curve that was fitted by a five-parameter logistic (5PL) curve-fitting model within SoftMax Pro version 7.0.3 (Molecular Devices, San Jose, CA). The Luminex standard is calibrated against the WHO international standard (NIBSC code 20/136) for anti-SARS-CoV-2 IgG antibodies, and results are reported in binding antibody units per milliliter (BAU/mL). The Luminex standard was also calibrated to the WHO international standard for anti-MERS-CoV IgG (NIBSC 19/178), and the MERS S assay result is reported in IU/mL. SARS-CoV-1 S, OC43 S, 229E S, HKU1 S, and NL63 S coronavirus protein results are reported as AU/mL.

### Sensitivity and specificity.

The sensitivity and specificity of the multiplex assay was evaluated with a set of known SARS-CoV-2-seropositive and -seronegative samples. A total of 160 serum and plasma samples, which were collected prior to December 2019, were selected for testing as SARS-CoV-2-seronegative samples to evaluate assay specificity. Sixty serum samples, which were collected from participants who were infected with and recovered from SARS-CoV-2, were selected for testing as SARS-CoV-2-seropositive samples to evaluate assay sensitivity. Positive predictive value (PPV) and negative predictive value (NPV) were also evaluated and estimated using combined PPA and combined NPA, respectively.

### Assay validation.

We developed an assay validation plan to evaluate the quantitative performance of the multiplex assay. A list of the parameters evaluated and brief details on the assay set up for each parameter is further described in Table S4 in the supplemental material. The upper and lower limits of quantitation were evaluated with 8 serum samples selected from SARS-CoV-2-infected participants. The samples were serially diluted and tested in duplicate by two analysts across 3 days. The acceptance criteria for the upper and lower limits of quantitation were as follows: CV ≤30% and percent error ≤50% at a given dilution. The geometric mean of the lowest concentration for each sample that passed the acceptance criteria was defined as the lower limit of quantitation, and the geometric mean of the highest concentration for each sample that passed the acceptance criteria was defined as the upper limit of quantitation. We determined intra- and interassay precision (CV) using 24 serum samples from SARS-CoV-2-infected or -vaccinated individuals spanning the assay range. The samples were serially diluted and tested in duplicate by two analysts across 5 days. Intra- and interassay precision acceptance criteria were at or below 25%. Accuracy was assessed with a single serum sample (SARS-CoV-2-infected participant) that was evaluated at three different antibody response levels. The sample was serially diluted and tested in five replicates by two analysts across 3 days. The acceptance criterion for accuracy was a calculated percent error at or below 25%. Linearity was formally assessed with four serum samples (SARS-CoV-2 infected participants). The samples were serially diluted and tested in quadruplicate by one analyst across 2 days. The acceptance criteria for linearity was as follows: CV ≤30% and percent error ≤50% at a given dilution. Finally, the cutoff (positive/negative threshold) was determined based on the mean concentration plus five standard deviations of 160 SARS-CoV-2-negative serum samples (collected before December 2019).

### Data analysis.

Luminex data (median fluorescence intensity) were exported from the instrument as CSV files. Bio-Plex Manager software v.6.2 (Bio-Rad, Hercules, CA) was used to convert the CSV file to 96-well plate formats. IgG concentrations (BAU/mL, IU/mL, or AU/mL as applicable to the protein) were determined by back calculations to the standard curves using a 5PL curve-fitting model within SoftMax Pro v.7.0.3. Results were exported as Excel worksheets, and data were analyzed using Microsoft Excel for Windows and Statistical Analysis System (SAS code). Prism v9.0 (GraphPad Software, San Diego, CA) was employed to generate plots.
