# Supplementary material for: Development, Validation, and Utilization of a Luminex-Based SARS-CoV-2 Multiplex Serology Assay
Source: Microbiol Spectr. 2023 Mar 16;11(2):e03898-22. doi: 10.1128/spectrum.03898-22 (PMC10100979; doi:10.1128/spectrum.03898-22)
Supplement: Supplemental file 1 — Supplemental material. Download spectrum.03898-22-s0001.docx, DOCX file, 0.7 MB [file spectrum.03898-22-s0001.docx]

**Supplemental Material: Figures and Tables**

**Supplementary Table 1: Comparison of Singleplex with Multiplex Assays.** Each comparison passed if the percent error was ≤ 25% between the two (geometric means of replicates were used if there was more than one replicate). The total number of samples, Relative Standard Deviation (RSD) %, Geometric Mean Ratio (GMR), and the percentage of total samples with percent error ≤ 25% (passing threshold) are given.

| **Assay*** | **Total Samples** | **RSD (%)** | **GMR** | **Passing (%)** |
| --- | --- | --- | --- | --- |
| CoV2 S | 8 | 3.7 | 1.030 | 87.5 |
| CoV2 N | 8 | 4.6 | 0.938 | 100 |
| CoV1 S | 8 | 6.9 | 0.932 | 87.5 |
| MERS S | 8 | 8.9 | 0.895 | 100 |
| OC43 S | 8 | 8.3 | 0.998 | 100 |
| 229E S | 8 | 5.9 | 1.031 | 100 |
| HKU1 S | 8 | 7.5 | 1.084 | 100 |
| NL63 S | 8 | 7.0 | 1.110 | 87.5 |
| RBD WT (R) | 8 | 5.5 | 1.083 | 75 |
| RBD N501Y mutant (R) | 8 | 2.7 | 1.033 | 100 |
| RBD E484K mutant (R) | 8 | 6.7 | 1.100 | 100 |
| RBD WT (M) | 8 | 3.1 | 1.046 | 75 |
| RBD N501Y mutant (M) | 8 | 8.2 | 1.122 | 100 |
| RBD triple mutant (M) | 8 | 4.9 | 1.054 | 100 |
| RBD E484K mutant (M) | 8 | 5.2 | 1.047 | 100 |

*- Protein sequences associated with Mount Sinai (M) and Ragon (R).

**Supplementary Figure 1:** Comparison of mean IgG results between the 15-plex and singleplex Luminex assays. Protein sequences associated with Mount Sinai (M) and Ragon (R). Note: the concentration units for MERS S is IU/mL.


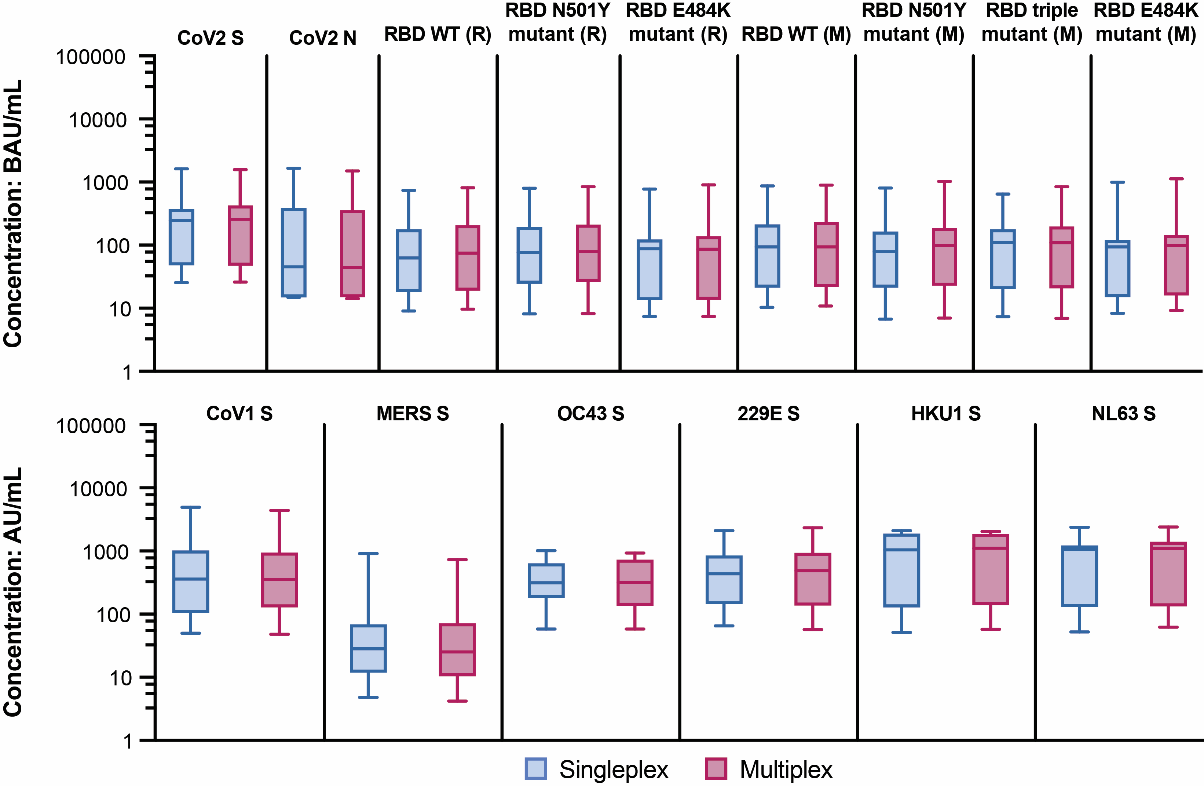


**Supplementary Table 2:** MFI and percent reduction of IgG signal in serum collected from participants (who were infected with and recovered from SARS-CoV-2) in the presence of SARS-CoV-2 S and CoV OC43 S proteins are shown for specificity assessment.

| **MFI: Signal** | **SARS-CoV-2 S** | **CoV-OC43** | **CoV-229E** | **SARS-CoV-1 S** |
| --- | --- | --- | --- | --- |
| Control: Diluent alone | 6046 | 40814 | 23020 | 4194 |
| SARS-CoV-2 S depleted | 949 | 41732 | 22113 | 422 |
| CoV OC43 S depleted | 5603 | 7788 | 22569 | 2535 |
|  | % Depletion (1-Specific signal/signal observed with control condition) | | | |
| SARS-CoV-2 S depleted | 84% | -2.2% | 3.9% | 90% |
| CoV OC43 S depleted | 7.3% | 81% | 2.0% | 40% |

**Supplementary Figure 2:** SARS-CoV-2 and non-CoV-2 proteins were tested with monoclonal antibodies from **(A)** BEI Resources Repository/Vaccine Research Center, NIAID, Manassas, VA, USA [RBD-binding mAb109, S2-binding mAb112, and mAb118] and **(B)** Sino Biologicals, Wayne, PA, USA [SARS-CoV/ CoV-2 S mAb1: 40150-D001, SARS-CoV/ CoV-2 S mAb2: 40150-D003, and SARS-CoV/ CoV-2 N mAb: 40143-R001]. The x-axis represents the protein, and the y-axis is the MFI value of the reactivity. Protein sequences associated with Mount Sinai (M) and Ragon (R).

**
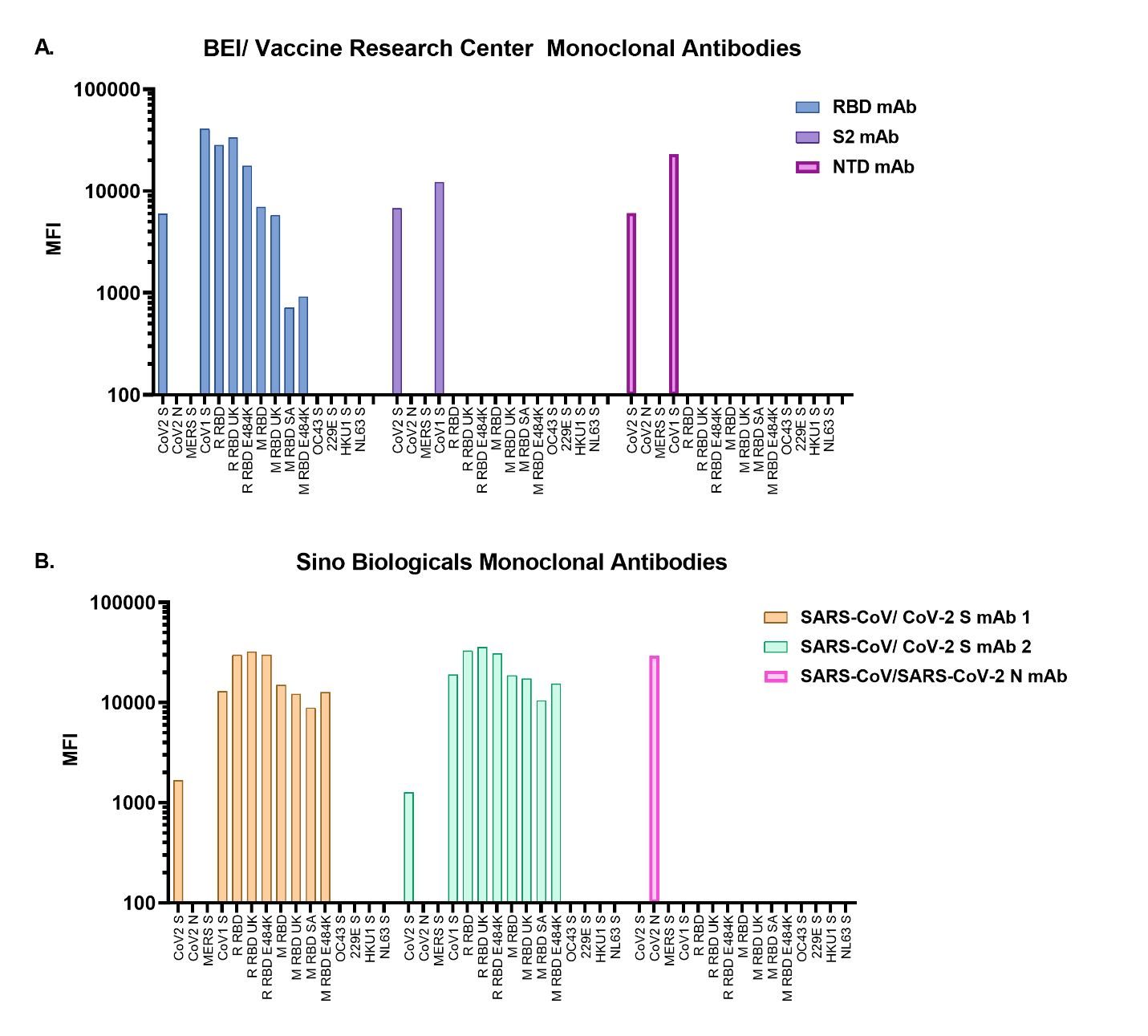
**

**Supplementary Table 3:** Optimized conditions for protein concentrations, secondary antibody concentration, incubation times, and buffer are given.

| **Parameter** | **Optimal Condition** |
| --- | --- |
| Capture Proteins Concentration | SARS-CoV-2, CoV-1, MERS CoV, Seasonal CoV S proteins: 5 μg/1.25 × 10^6^ beads  SARS-CoV-2 RBD proteins: 5 μg/1.25 × 10^6^ beads  SARS-CoV-2 N protein: 0.5 μg/1.25 × 10^6^ beads |
| Sample Incubation Time | 30 minutes incubation time at RT |
| PE-Conjugated IgG Antibody Concentration | 125-fold diluted from stock |
| Pre-Coupled Beads Storage Buffer & Assay Diluent | Dulbecco’s PBS with 1% BSA & 0.2 % ProClin |

**Supplementary Table 4.** Assay Validation Analysis Procedures and Acceptance Criteria

| Parameter | Samples (n) | Replicates | Analyst(s) | Days (Runs) | Methodology | Acceptance Criteria |
| --- | --- | --- | --- | --- | --- | --- |
| Standard Curve | 8-point dilution | 2 | 3 | 91 | Calculate back calculated concentration, RSD, and R^2^ | RSD for standards that span LLOQ and ULOQ ≤ 30%; R^2^ ≥ 0.90 |
| Controls | 4-point dilutions of high, mid, and low-level controls | 1 | 3 | 91 | Calculate Geometric Mean, RSD, and accuracy for each dilution | RSD: ≤30%  Accuracy: At least 2/3 of the control measurements should meet 70-130% accuracy. |
| LOQ | 8 | 2 | 2 | 3 | Calculate Geometric Mean, RSD, and Percent Error for each dilution | Percent Error ≤ 50%; RSD ≤ 30% |
| Accuracy | 3 | 5 | 2 | 3 | Calculate Geometric Mean and Percent Error | Percent Error ≤ 25% |
| Precision | 24 | 2 | 2 | 5 | Calculate Geometric Mean and RSD for Intra-plate, Inter-plate, and Inter-Analyst | RSD ≤ 25% for Intra-plate, Inter-plate, and Inter-Analyst |

**Supplementary Table 5.** List of the MFI Values and Concentrations for the Luminex Standard for each of the 15 CoV Proteins.

|  | **CoV-2 S** | | **CoV-2 N** | | **CoV-1 S** | | **MERS CoV S** | | **OC43 CoV S** | | **229E CoV S** | | **HKU1 CoV S** | | **NL63 CoV S** | |
| --- | --- | --- | --- | --- | --- | --- | --- | --- | --- | --- | --- | --- | --- | --- | --- | --- |
| **Standard** | **Conc. (BAU/mL)** | **MFI** | **Conc. (BAU/mL)** | **MFI** | **Conc. (AU/mL)** | **MFI** | **Conc. (IU/mL)** | **MFI** | **Conc. (AU/mL)** | **MFI** | **Conc. (AU/mL)** | **MFI** | **Conc. (AU/mL)** | **MFI** | **Conc. (AU/mL)** | **MFI** |
| **1** | 44.2 | 47166 | 13.5 | 41061 | 50 | 14569 | 9.3 | 10903 | 50 | 49296 | 50 | 24306 | 50 | 21231 | 50 | 13901 |
| **2** | 14.7 | 41095 | 4.5 | 43054 | 16.7 | 7815 | 3.1 | 8422 | 16.7 | 40229 | 16.7 | 14287 | 16.7 | 10860 | 16.7 | 7034 |
| **3** | 4.91 | 29318 | 1.5 | 37052 | 5.56 | 3853 | 1.03 | 5502 | 5.56 | 25992 | 5.56 | 8015 | 5.56 | 5218 | 5.56 | 3475 |
| **4** | 1.64 | 16247 | 0.5 | 22871 | 1.85 | 1753 | 0.344 | 2578 | 1.85 | 13300 | 1.85 | 3552 | 1.85 | 2111 | 1.85 | 1418 |
| **5** | 0.546 | 8157 | 0.167 | 11782 | 0.617 | 765 | 0.115 | 1130 | 0.617 | 5799 | 0.617 | 1439 | 0.617 | 797 | 0.617 | 563 |
| **6** | 0.182 | 3378 | 0.0556 | 5129 | 0.206 | 322 | 0.0383 | 436 | 0.206 | 2507 | 0.206 | 578 | 0.206 | 323 | 0.206 | 233 |
| **7** | 0.0606 | 1444 | 0.0185 | 2170 | 0.0686 | 123 | 0.0128 | 172 | 0.0686 | 959 | 0.0686 | 226 | 0.0686 | 126 | 0.0686 | 107 |
| **8** | 0.02 | 586 | 0.006 | 827 | 0.023 | 62 | 0.004 | 76 | 0.023 | 349 | 0.023 | 94 | 0.023 | 61 | 0.023 | 54 |

|  | **RBD WT (R)** | | **RBD mutant N501Y (R)** | | **RBD mutant E484K (R)** | | **RBD WT (M)** | | **RBD mutant N501Y (M)** | | **RBD triple mutant (M)** | | **RBD mutant E484K (M)** | |
| --- | --- | --- | --- | --- | --- | --- | --- | --- | --- | --- | --- | --- | --- | --- |
| **Standard** | **Conc. (BAU/mL)** | **MFI** | **Conc. (BAU/mL)** | **MFI** | **Conc. (BAU/mL)** | **MFI** | **Conc. (BAU/mL)** | **MFI** | **Conc. (BAU/mL)** | **MFI** | **Conc. (BAU/mL)** | **MFI** | **Conc. (BAU/mL)** | **MFI** |
| **1** | 16.5 | 39318 | 23.5 | 37827 | 12.5 | 35765 | 19 | 36904 | 20.1 | 35491 | 19.4 | 17973 | 14.1 | 25855 |
| **2** | 5.5 | 33851 | 7.83 | 35708 | 4.17 | 23076 | 6.33 | 31141 | 6.7 | 30149 | 6.47 | 9868 | 4.7 | 14093 |
| **3** | 1.83 | 23987 | 2.61 | 26607 | 1.39 | 12126 | 2.11 | 20069 | 2.23 | 19226 | 2.16 | 4343 | 1.57 | 6451 |
| **4** | 0.611 | 12648 | 0.87 | 15746 | 0.463 | 5093 | 0.704 | 10082 | 0.744 | 9741 | 0.719 | 1591 | 0.522 | 2482 |
| **5** | 0.204 | 5869 | 0.29 | 7608 | 0.154 | 2106 | 0.235 | 4394 | 0.248 | 4232 | 0.24 | 617 | 0.174 | 911 |
| **6** | 0.0679 | 2453 | 0.0967 | 3381 | 0.0514 | 823 | 0.0782 | 1601 | 0.0827 | 1660 | 0.0798 | 226 | 0.058 | 332 |
| **7** | 0.0226 | 916 | 0.0322 | 1344 | 0.0171 | 322 | 0.0261 | 597 | 0.0276 | 695 | 0.0266 | 95 | 0.0193 | 130 |
| **8** | 0.008 | 353 | 0.011 | 543 | 0.006 | 132 | 0.009 | 225 | 0.009 | 297 | 0.009 | 46 | 0.006 | 58 |
